# Characterizing socially avoidant and affiliative responses to social exclusion

**DOI:** 10.3389/fnint.2012.00046

**Published:** 2012-07-10

**Authors:** Katherine E. Powers, Todd F. Heatherton

**Affiliations:** Department of Psychological and Brain Sciences, Dartmouth College, HanoverNH, USA

**Keywords:** social exclusion, need to belong, withdrawal, affiliation, social brain, self-esteem, medial prefrontal cortex, ventral anterior cingulate cortex

## Abstract

Humans have a fundamental need for social relationships. From an evolutionary standpoint, the drive to form social connections may have evolved as an adaptive mechanism to promote survival, as group membership afforded the benefits of shared resources and security. Thus, rejection from social groups is especially detrimental, rendering the ability to detect threats to social relationships and respond in adaptive ways critical. Previous research indicates that social exclusion alters cognition and behavior in specific ways that may initially appear contradictory. That is, although some studies have found that exclusionary social threats lead to withdrawal from the surrounding social world, other studies indicate that social exclusion motivates affiliative social behavior. Here, we review the existing evidence supporting accounts of avoidant and affiliative responses, and highlight the conditions under which both categories of responses may be simultaneously employed. Then, we review the neuroimaging research implicating specific brain regions underlying the ability to detect and adaptively respond to threats of social exclusion. Collectively, these findings are suggestive of neural system highly attuned to social context and capable of motivating flexible behavioral responses.

## Introduction

Humans have a fundamental need for social groups (Bowlby, 1969; Baumeister and Leary, 1995). From an evolutionary perspective, group membership affords the benefits of shared resources and security. Because social exclusion poses critical challenges for survival, the drive to maintain social relationships may have evolved for adaptive purposes. As a basic human motive, the need to belong activates behavior and influences cognition and emotion. Failure to satisfy this need for close social connections has been associated with a variety of adverse consequences, including self-defeating behaviors, negative moods, and mental and physical health complications (Twenge et al., 2001; Cacioppo et al., 2006).

The need to belong theory is supported by evidence that people feel anxious when facing actual or potential exclusion from social groups (Baumeister and Tice, 1990). According to social exclusion theory, people are socially excluded for reasons of immorality, incompetence, or unattractiveness. Breaking group norms and rules, which is the essence of immorality, threatens group structure; incompetence provides a drain on group resources; and being physically unattractive or having a stigmatizing condition may suggest inferior genes.

In order to induce experiences of social exclusion in the laboratory, researchers have utilized a variety of manipulations, including playing the virtual ball-tossing game Cyberball, receiving fictitious predictions that their future lives will be isolated and lonely, and recalling past experiences of rejection (for comprehensive reviews of the various methodologies, we point interested readers to Baumeister et al., 2007, and DeWall et al., 2011a). Results of previous behavioral research indicate that experiencing social exclusion alters cognition and behavior in specific ways that may initially appear contradictory. That is, although some studies have found that social exclusion leads to withdrawal from the surrounding social world and emotional numbness (e.g., Twenge et al., 2003; Baumeister et al., 2007), other studies suggest that social exclusion actually motivates affiliative social behavior (e.g., Gardner et al., 2000; Pickett et al., 2004; Maner et al., 2007). Here, we review the existing evidence supporting both avoidant and affiliative responses, and highlight the conditions under which both categories of responses may be simultaneously employed. Furthermore, given the importance of group inclusion, there ought to be mechanisms for detecting as well as adaptively responding to threats of social exclusion (Heatherton, 2011). After reviewing the behavioral findings, we discuss the neuroimaging research implicating the specific brain regions underlying the ability to detect and adaptively respond to threats of social exclusion.

## Avoidance of social information

Prior research suggests that social exclusion motivates withdrawing from the surrounding social world and produces feelings of emotional detachment (Twenge et al., 2003; DeWall and Baumeister, 2006; Baumeister et al., 2007). Specifically, Twenge et al. (2003) demonstrated that social exclusion results in lethargy, avoidance of self-awareness, and emotional numbness. Additional support for emotional numbness following exclusion is abundant, as excluded participants repeatedly fail to report occurrences of negative mood or aversive emotions (e.g., Twenge et al., 2001, 2002, 2003, 2007; Baumeister et al., 2002; DeWall and Baumeister, 2006; Maner et al., 2007; DeWall et al., 2009). Moreover, when differences are found, they do not mediate the observed behavioral effects, suggesting that experiencing social exclusion results in a state of emotional numbness, rather than acute emotional distress. Withdrawal and emotional numbness may help avoid the initial pain of an experience of rejection, as well as protect the self from further experiences of social distress (Baumeister et al., 2007).

One consequence of this withdrawal is the apparent failure to demonstrate concern for others. Following receiving fictitious feedback about having a lonely future life, participants donated less money to student funds, expressed disinterest in volunteering for future lab experiments, and picked up fewer dropped pencils (Twenge et al., 2007). Moreover, social exclusion decreases motivation to attend to the emotional states of others. Indeed, excluded participants report less empathic concern for the social misfortunes of others, such as being rejected by a romantic partner (DeWall and Baumeister, 2006; Twenge et al., 2007).

Such a disinterest in other people and failure to consider their emotional states may even lead to aggressive interpersonal behaviors. Across several studies, excluded participants were more likely to provide negative job evaluations (Twenge et al., 2001), deliver an aversive noise (Twenge et al., 2001), administer excessive amounts of hot sauce to participants who dislike spicy food (DeWall et al., 2010), and force others to listen to an annoying tape (Buckley et al., 2004).

On a broader level, these responses may represent failures of regulatory efforts to exert self-control (Baumeister et al., 2007). The capacity to properly regulate behavior and control impulses, and to put the needs of the group above one's own, is critical for maintaining social relationships and group cohesion (Heatherton, 2011). From this perspective, effectively regulating behavior is closely related to social acceptance, as failure to do so may lead to undesirable outcomes, such as eviction from social groups (Twenge et al., 2001; DeWall et al., 2011a). Several studies have further probed this link by investigating how social exclusion directly affects self-regulatory performance. Exclusion appears to impair self-regulatory efforts, as measured by decreased performance on intelligence tests and tasks requiring executive function (Baumeister et al., 2002, 2005) and an increased tendency to eat unhealthful foods (Baumeister et al., 2005). It should be noted that providing additional motivations to regulate behaviors (e.g., offering a cash incentive) reverses these effects, suggesting that exclusion renders people unwilling, but not unable, to exert self-regulatory control (DeWall et al., 2011a). Thus, the impaired regulatory performance described above suggests that excluded individuals may simply not care about gaining positive impressions (and ultimately, social acceptance) from others.

In summary, experiencing social exclusion has been shown to result in withdrawal and emotional numbness. Consequently, people have a diminished desire to empathize with others, occasionally even engaging in aggressive behaviors. These behavioral tendencies are consistent with the reasoning that exclusion leads to a lack of concern for others, resulting in reduced motivation to regulate behavior in desirable ways and obtain social acceptance.

## Attention to social information

In contrast to the withdrawal pattern, other work suggests that social exclusion alters cognition and behavior in more socially affiliative ways. Notably, social exclusion appears to bias cognitions such that people can more readily identify social information (see also Cacioppo and Cacioppo, 2012). Gardner et al. (2000) investigated the saliency of social information following social exclusion. Participants engaged in a simulated chat room experience during which they were accepted or rejected by supposed peers. Following this experience, participants read diary entries containing social and nonsocial information, and were administered a surprise memory test for events in the diaries. Results revealed that socially excluded participants displayed enhanced memory for social information and events. Similarly, Pickett et al. (2004) provided evidence that the desire to have a lot of social relationships heightens attention paid to the surrounding social world. Specifically, people who reported having a strong desire to belong to social groups demonstrated greater accuracy in identifying emotional facial expressions, as well as the valence of spoken words.

Just as important as identifying social information is determining its authenticity. Indeed, socially excluded people can more readily distinguish between real and fake (“Duchenne”) smiles (Bernstein et al., 2008) and display a preference for real smiles (Bernstein et al., 2010), suggesting a heightened ability to decode social information.

These studies offer the possibility that social information becomes more salient following social exclusion because it signals potential affiliation opportunities. In line with this reasoning, Maner et al. (2007) demonstrated that social exclusion leads people to view others in a more positive light (e.g., as more friendly and desirable) and to display an increased desire to work with others in groups rather than working alone. Such biased cognitions may generalize to generally seeking out more positive stimuli. Indeed, DeWall et al. (2011b) demonstrated that excluded participants spontaneously recalled more positive events than those who did not experience exclusion. Moreover, exclusion leads people to group words together based on positive emotional connotations (e.g., matching “puppy” with “parade” instead of “beetle”), and to complete more word stems with words depicting positive emotions.

Additional research has investigated the overt behavioral responses resulting from these cognitive biases. Specifically, excluded people appear motivated to engage in affiliative social behavior. Maner et al. (2007) performed a series of studies examining behavior following manipulations of social exclusion. They found that social exclusion leads to a greater desire to make new friends and form social bonds, and to work cooperatively with others on tasks.

Together, this research illustrates how social exclusion can bias cognitive processes in socially affiliative ways. These behaviors are likely driven by the desire to re-establish social bonds, and ultimately, may increase the likelihood of gaining acceptance from others (DeWall et al., 2011a).

## Simultaneously employed processes

For years, these seemingly contradictory accounts of responses to social exclusion prevailed. However, recent evidence suggests that responses to social exclusion may be more nuanced than simply avoiding or approaching others. Instead, people might simultaneously employ both defensive and affiliative strategies, allowing them to avoid further distress while also encouraging the establishment of positive social connections (Hess and Pickett, 2010). This line of reasoning is supported by an eye-tracking study by DeWall et al. (2009) in which socially excluded participants displayed decreased attention to negative social stimuli while selectively attending to signs of social acceptance. Moreover, exclusion results in differential attempts to infer the mental states of others. Specifically, people apparently display a preference for mentalizing about positive social information and avoid considering negative aspects of their social world (Powers et al., in press).

This interpretation converges nicely with one of the earliest models of self-regulation (Carver and Scheier, 1982), in which people regulate their behaviors in adaptive and profitable ways when favorable outcomes are expected, but escape from self-awareness and withdraw when unfavorable outcomes are expected. Indeed, unfavorable outcomes following social exclusion (e.g., thinking about the mental states of potential social threats) are met with mental withdrawal, while favorable outcomes (e.g., re-establishing social ties) are met with continued, possibly enhanced efforts.

## Social exclusion and self-esteem

If humans have a fundamental need to belong, then there ought to be dedicated mechanisms for detecting threats to social inclusion (Leary et al., 1995; Heatherton, 2011). Put another way, given the fundamental importance of group inclusion to mental and physical health, humans need to be especially sensitive to signs that the group might exclude them. According to the sociometer model, self-esteem functions as a monitor of the status of interpersonal relationships and the possibility of social exclusion. When people behave in ways that increase the likelihood they will face exclusion, they experience a reduction in state self-esteem (Leary et al., 1995).

According to the sociometer theory, people likely vary in terms of how their sociometers are calibrated (Leary et al., 1995). For instance, high self-esteem individuals generally feel accepted and included and expect others to like them. Therefore, they may be less concerned with interpersonal evaluation than people with moderate or low self-esteem (Leary and Downs, 1995). Indeed, when asked to estimate occurrences of positive and negative feedback, individuals with high self-esteem reported receiving more positive feedback than those with low self-esteem. Moreover, those with high self-esteem consistently overestimated the amount of positive feedback they received, while those with low self-esteem were generally accurate in their estimations (Somerville et al., 2010b). Although people with high self-esteem apparently do experience a reduction in feelings of state self-esteem when excluded, they may not drop to a level that suggests they are in imminent danger of being excluded. By contrast, people with low self-esteem have a tendency to more readily perceive rejection, and this is reflected in the relative calibration of their sociometers (Leary et al., 1995).

## Associated brain mechanisms

Given the importance of group inclusion for survival, various brain mechanisms may be particularly attuned to information about the social world. From this perspective, there ought to be neural mechanisms for detecting threats of social exclusion, as well as adaptively responding to them (Heatherton, 2011). Indeed, neuroimaging research has revealed that specific brain regions support these dissociable processes.

Studies examining neural responses during the experience of social exclusion have consistently revealed activation in the anterior cingulate cortex (ACC), although some studies implicate more dorsal ACC regions (e.g., Eisenberger et al., 2003), while others observed more ventral activations (e.g., Somerville et al., 2006). The ACC is involved in a variety of cognitive and affective processes (see Bush et al., 2000), and debate in the field still exists regarding the exact region of the ACC involved in detecting threats of social exclusion; however we note the extant literature that implicates ventral ACC in emotional and social processes and disorders including depression (Drevets et al., 1997; Mayberg et al., 2005; Moran et al., 2006). Other brain regions that have been shown to respond to social exclusion include the ventrolateral prefrontal cortex (vlPFC), which may be involved in the regulation of distress, and the insula, which has been implicated in processing the sensory components of physical pain (Eisenberger et al., 2003; Eisenberger and Lieberman, 2005).

During adolescence, the salience of peer interactions and an increased desire to be accepted by others suggest that the brain may uniquely respond to experiences of social exclusion (Somerville et al., 2010a; Masten et al., 2011). Masten et al. (2009) scanned adolescents during an experience of social exclusion and found activation in the insula. However, they noted that experiencing social exclusion failed to recruit the dorsal ACC or vlPFC, as has been previously reported in adults. Other studies have consistently revealed involvement of the ventral ACC in adolescents experiencing social exclusion (Gunther Moor et al., 2010; Sebastian et al., 2011). Taken together, neural responses in adolescents to social exclusion appear to be marked by engagement of the ventral ACC and insula. That the vlPFC is not implicated in adolescents as it is in adults is may be indicative of a differential sensitivity to social exclusion across the lifespan (Pfeifer and Blakemore, 2012). Alternatively, the ongoing maturation of the PFC during adolescence may lead to activation patterns that would differ from that of adults.

To the extent that self-esteem functions as a monitor of the likelihood of social exclusion, this ought to be reflected by differential neural responses. Somerville et al. (2010b) examined functional brain activity in response to evaluative social feedback as a function of self-esteem. They found that activity within ventral ACC is modulated by self-esteem, such that individuals with low self-esteem display enhanced activity to positive versus negative feedback. Ventral ACC activity did not distinguish between positive and negative feedback for individuals with high self-esteem. This finding suggests a neural mechanism underlying the particular sensitivity of individuals with low self-esteem to cues indicative of social standing, and further implicates the ventral ACC as critical in the representation of social relations.

**Figure 1:**
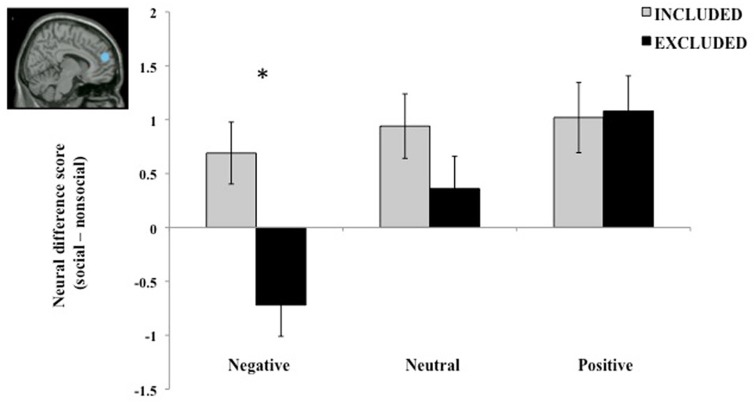
**Following social exclusion, activity in dmPFC, a brain region involved in mentalizing, (1) is reduced when viewing negative social scenes, and (2) increases in a linear fashion across valence categories (from negative to neutral to positive).** Inset displays location of dmPFC ROI (6, 54, 21). Thus, people may be motivated to mentalize about the positive aspects of their social worlds following rejection, but avoid doing so for negative social information (Powers et al., in press).

This work highlights the neural mechanisms involved in detecting threats of social exclusion. Recently, in order to more clearly understand the cognitive processes underlying the differential behavioral reactions to social exclusion detailed above, we explored neural responses immediately following an experience of social exclusion (Powers et al., in press). We employed a modified version of a social exclusion manipulation used in previous behavioral research (see Twenge et al., 2001), in which participants were provided with fictitious feedback indicating that their futures would be filled with long-lasting, stable relationships (social inclusion) or that they would be isolated and lonely (social exclusion). Participants were then scanned while viewing a series of pictures varying in social (i.e., with people, without people) and emotional (i.e., negative, neutral, positive) content. We found that socially excluded individuals failed to recruit dorsomedial prefrontal cortex (dmPFC), a brain region consistently implicated in mentalizing, for negative social scenes. Critically, dmPFC was still engaged when viewing positive social scenes. Moreover, following social exclusion, dmPFC demonstrated a linear effect of valence, with greater activity to positive social scenes compared to negative social scenes (see Figure 1). Importantly, there was no effect of social exclusion on dmPFC response to nonsocial scenes. Our results suggest that people are motivated to mentalize about the positive aspects of their social worlds following rejection, but avoid doing so for negative social information. Thus, the behavioral strategies engaged in response to social exclusion may reflect differential engagement of brain regions involved in understanding the mental states of others.

Taken together, this evidence suggests that neural activity differs depending on whether people are actively being excluded or responding to a very recent experience. While open questions remain, as neuroimaging research in this field is still in its infancy, these findings do highlight the sensitivity of the brain to social context and the status of interpersonal relationships, and offer insight into developmental changes.

## Summary

Emotional threats can enhance or impair social cognition. Responses to social exclusion seem to engender categorically oppositional reactions to socially relevant stimuli and situations, with research showing both that social exclusion motivates withdrawal from the surrounding social world and antisocial behaviors and also that excluded people appear highly attuned to social information, specifically that which is positive, and display a propensity to engage in prosocial behaviors. That is, people may concurrently employ avoidant and affiliative strategies in an effort to most adaptively respond to social threats. In this way, people may protect themselves from further distress while simultaneously attempting to form positive social connections with others. Neuroimaging research has revealed that specific neural regions, notably the ACC and mPFC, support the ability to detect and adaptively respond to social threats. Across these responses, a clear pattern of social specificity emerges. That is, converging behavioral and neuroimaging evidence reveals that both affiliative and avoidant responses to social exclusion are specific to social stimuli. Considered in concert, these findings are suggestive of neural system highly attuned to social context and capable of motivating flexible behavioral responses.

### Conflict of interest statement

The authors declare that the research was conducted in the absence of any commercial or financial relationships that could be construed as a potential conflict of interest.
